# Symptoms and adverse events in controlled human infection models

**DOI:** 10.3389/fmed.2025.1578560

**Published:** 2025-08-14

**Authors:** Karen Götz, Poleta Luga, Jörg Rengel, Mei Masur, Marcela Juárez-Hernández, Isabelle Bekeredjian-Ding

**Affiliations:** ^1^Division of Infectious Disease, Paul-Ehrlich-Institut, Langen, Germany; ^2^Institute for Medical Microbiology and Hospital Hygiene and Regulatory Intelligence Team, Philipps-University Marburg, Marburg, Germany; ^3^German Center for Infection Research (DZIF), Partner Site Giessen-Marburg-Langen, Marburg, Germany

**Keywords:** human challenge trials, CHIM, vaccine, anti-infective, adverse effects, safety

## Abstract

The potential and positioning of controlled human infection models (CHIMs) and human challenge trials (HCTs) in the investigation of infectious pathogens and efficacy of new anti-infectives or vaccines are under evaluation. CHIMs and HCTs can provide supporting data for decision-making in the development of new medicines (“fast failure”). However, it is important to consider that, like in any phase 1 trial, CHIM volunteers have no direct health benefit. Approval by an ethics or regulatory board implies cautious evaluation of risk and potential safety issues. In this study, we chose a syndromic approach to summarize CHIM and HCT adverse events (AEs). AEs were grouped by disease entities, e.g., enteric, respiratory, vector-borne, and parasitic infections. The analysis concludes that severe AEs are rare. It confirms that AEs reflect symptoms of CHIM infections and are less prevalent in CHIM intended for the induction of carriage. Furthermore, the number of subjects affected reflects the attack rate and individual predisposition. Rarely, AEs affect the study participants’ daily activities, ranging from impairing and preventing routine tasks to requiring emergency room visits or hospitalizations. Nevertheless, while AEs guide ethical and regulatory considerations, symptoms are needed as endpoints for evaluation of the efficacy of drugs or vaccines. Finally, we observe a lack of harmonization in the reporting and grading of AEs. This reveals an eminent need for a reporting structure that allows accessibility and comparability of data sets.

## Highlights

- Grouping of CHIM studies by disease entities identified disease group-specific symptoms, adverse events, and required medical interventions.
- Distinction of adverse events from vaccines or drugs from infection symptoms provoked by infectious challenge can be difficult.
- Medical interventions reduce disease-specific risks but mitigate specific symptoms and severity of infection in CHIM studies.
- Standardization of AEs reporting in CHIM studies should be sought to allow better comparison of study data and provide a better understanding of the risks.
- Communication on potential scientific and social value and risks is key to societal acceptance of CHIM studies.

## Introduction

In humanity’s fight against infections, infection control and sanitation measures have dramatically reduced transmission rates for many infectious pathogens over the last centuries. Concomitantly, the availability of vaccines and anti-infectives has made many infections treatable and preventable. Nevertheless, infections remain a frequent cause of death worldwide. This surges the clinical need for the development of new vaccines and anti-infectives. Contemporary challenges include the rise in antimicrobial resistance and the spread of zoonotic emerging viruses to humans.

In the past decades, controlled human infection models (CHIMs) have been established and evaluated for many pathogens. However, their value and positioning in drug and vaccine development have remained an ethical matter of debate fueled by historical misconduct (1, 2). Currently available CHIMs include the full range of bacterial, viral, and parasitic infections. These studies have been used to describe immune correlates of protection and for testing new medicines and vaccines for efficacy in so-called human challenge trials (HCTs). This can facilitate early decision-making in product development (“fast failure”) or the testing of medicines in specific populations, such as travelers at risk for infections.

Recently, significant efforts have been made to establish ethical guidelines for CHIM development and HCTs (1–4). Notably, the most relevant basis for decision-making on going forward with a challenge model is the assessment of risk versus benefit of potential results. Since these studies imply that healthy human subjects are intensively exposed to infection, it is necessary to carefully predict and evaluate the safety risks for these individuals. The most important principle is to avoid harm, “*primum non nocere*.” This implies that in order to minimize adverse events (AEs) in the study population, it is necessary to balance the severity of disease manifestation that serves as a clinical endpoint with safety considerations and to ensure the stability of the genotype and robustness of the phenotypical and functional characteristics of the challenge strains across trials (5–7). Thus, selection and specifications of challenge agents are key to an understanding of the present and future potential of CHIMs. Furthermore, when treatment is established, CHIM studies are considered feasible, but this safety measure is not always available, especially in diseases where high clinical need drives the search for new therapies and vaccines.

Despite multiple reports on CHIM and HCT outcomes and their positioning in decision-making (8, 9), only few, usually disease-specific reports specifically address safety issues in HCTs and CHIMs and attempt to define an acceptable residual risk for volunteers that could be used to generate disease- and pathogen-specific CHIM recommendations. This could arise from uncertainties in regard to the requirement for differentiation of infection symptoms and AEs, as well as procedure-related needs in CHIM strain selection, e.g., safety and acceptability versus infection requirements. A clearer and more specific AE definition in CHIMs and HCTs could, therefore, be beneficial. The objective of the present study was, thus, to provide a basis for an understanding of the attributable risks and acceptability thresholds as well as an improved informed consent. This could increase acceptance from both regulators and subjects. In this study, we provide an analysis and summary of AEs using a syndromic approach by grouping challenge agents by disease entity.

## Methods

### Literature search and selection

Studies were preselected based on PubMed searches for either ‘CHIM (or HCT) AND safety OR adverse events (AE)’. A second search retrieved articles from clinical databases and Google Scholar. The reports selected were peer-reviewed articles, published in English language, free full texts, and screened for duplicates. Due to language barriers, only studies in English could be included in the review. White papers were not added because they do not provide study data and are not peer-reviewed.

All reports were independently screened by two reviewers to ensure the consistency of the selection process. To ensure methodological rigor and credibility of our findings, gray (non-peer-reviewed) literature and unpublished or preprint data were excluded from this report. Studies were included based on coherent reporting of symptoms and AEs in predetermined disease entities, e.g., enteric, respiratory, vector-borne, and water- or soil-transmitted parasitic infections. Of note, this approach resulted in a limited but representative number of reports for analysis. Nevertheless, in view of the high number of publications in an emerging field, the authors cannot exclude that individual publications might not have been assessed. The present analysis summarizes the results obtained in 41 reports on CHIMs and HCTs published or re-analyzed after the year 2000 to provide a clear picture of the current practice. Notably, in some cases, reference is made to earlier studies to highlight the evolvement of the specific trials in regard to standardization and safety reporting. Symptoms and AEs documented in the studies were grouped by disease entity to provide a more general picture of the burden for participating volunteers during infection type-specific CHIMs. In the tables with summarized data, we included only studies that reported absolute numbers or percentages of subjects experiencing a defined AE; studies limited to “AEs recorded” without quantification were excluded. For HCTs, it was often more precise to refer to the placebo group instead of the total population. When applicable, this is denoted with (*) in all tables.

### Distinction of clinical symptoms and adverse events

In CHIMs and, in particular, in HCTs, there is an uncertainty and potentially an inherent overlap of AE and CHIM-inherent symptoms of infection that often remain unaddressed. The available non-binding recommendations and guidelines are neither suitable for distinguishing these nor do they provide guidance for precise and comparable pathogen or disease-specific grading. In many studies, it remained unclear whether AEs during CHIMs were potentially underreported or neglected by rating them as clinical endpoints (disease manifestation defined by a predetermined combination of symptoms) and according to which criteria AEs were graded from mild to potentially severe, life-threatening AEs. Severity grading was often based on study-specific rating scales such as symptom scorecards and pro-flu questionnaires, especially when other disease-relevant, evidence-based scales were not available or deemed inappropriate. Inconsistent severity grading prevents comparability and can lead to inaccurate interpretation. We, therefore, decided to summarize and report AEs after challenge without differentiating according to the diverging definitions and severity grading.

## Results

### Enteric infections with fecal-oral transmission

**Figure 1:**
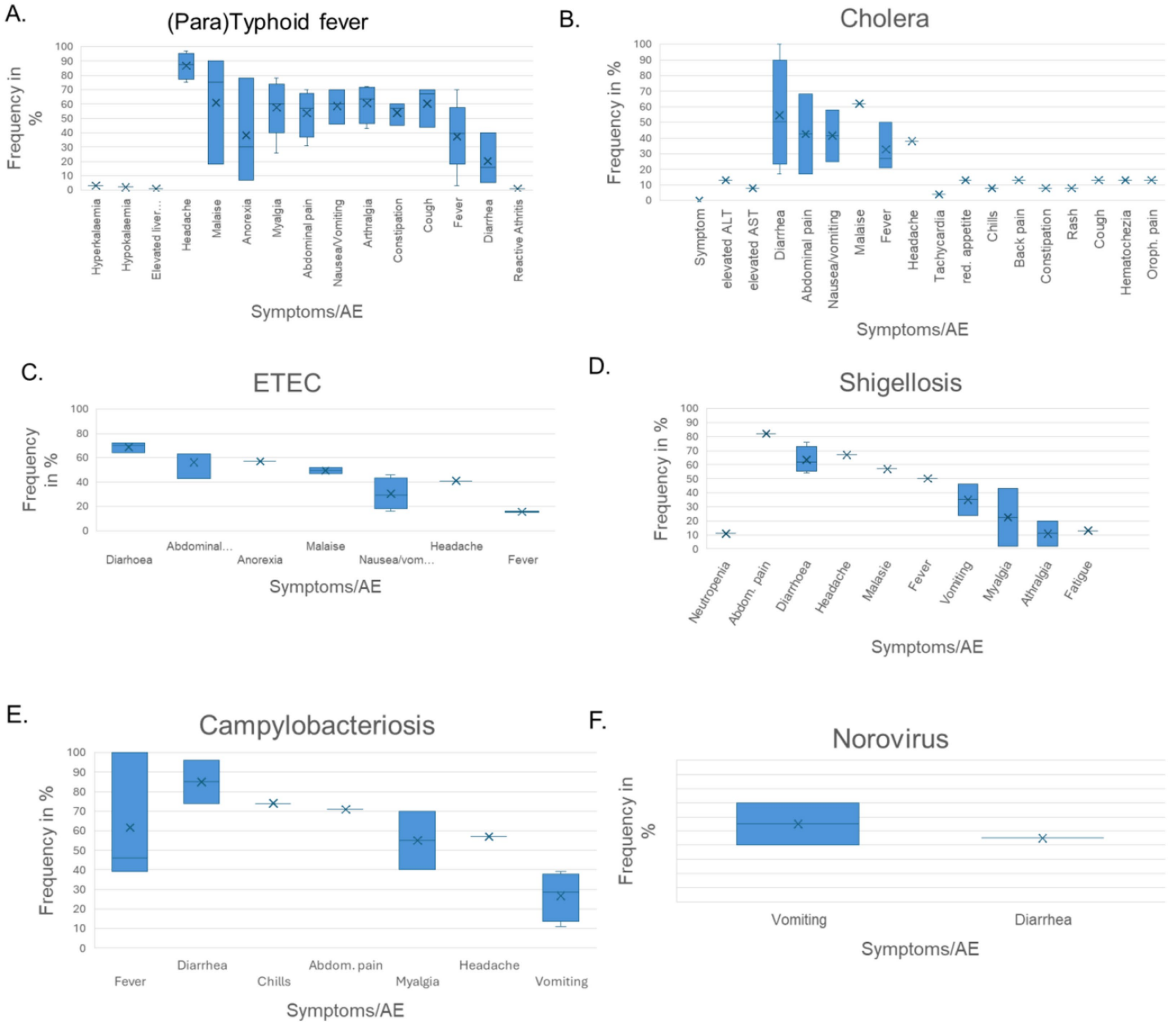
Graphical summary of symptom frequencies (AEs) in CHIMs for enteric infections. Reported AEs are given in percentages (%). **(A)** (Para)typhoid fever. **(B)** Cholera. **(C)** ETEC (enterotoxigenic *Escherichia coli*). **(D)** Shigellosis. **(E)** Campylobacteriosis. **(F)** Norovirus.

CHIMs have frequently been employed in the context of vaccine development against enteric pathogens such as typhoid and paratyphoid fever (10–13), cholera (14–16), enterotoxigenic *E. coli* (17–19), Shigella (20–22), *Campylobacter jejuni* (23, 24), and norovirus infections (25). Thus, we evaluated 15 reports on CHIMs describing symptoms (e.g., AEs) caused by bacterial diarrheal disease manifestation and *n* = 1 on viral (norovirus) infection. A detailed summary of AEs categorized by CHIMs is shown in Tables 1–6. with references and summarized in Figure 1.

**Table 1:**
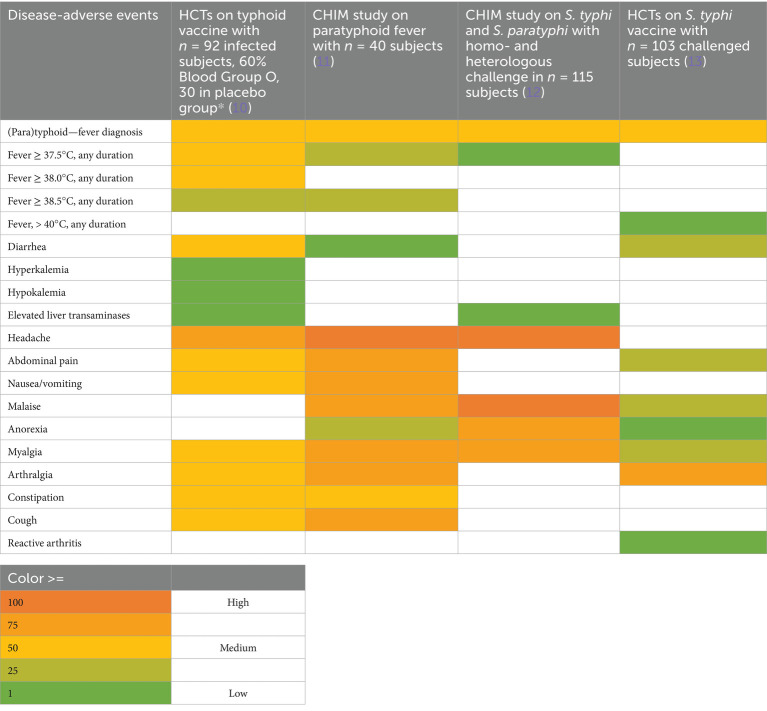
Adverse events in CHIMs for enteric infections—(para)typhoid.

**Table 2:**
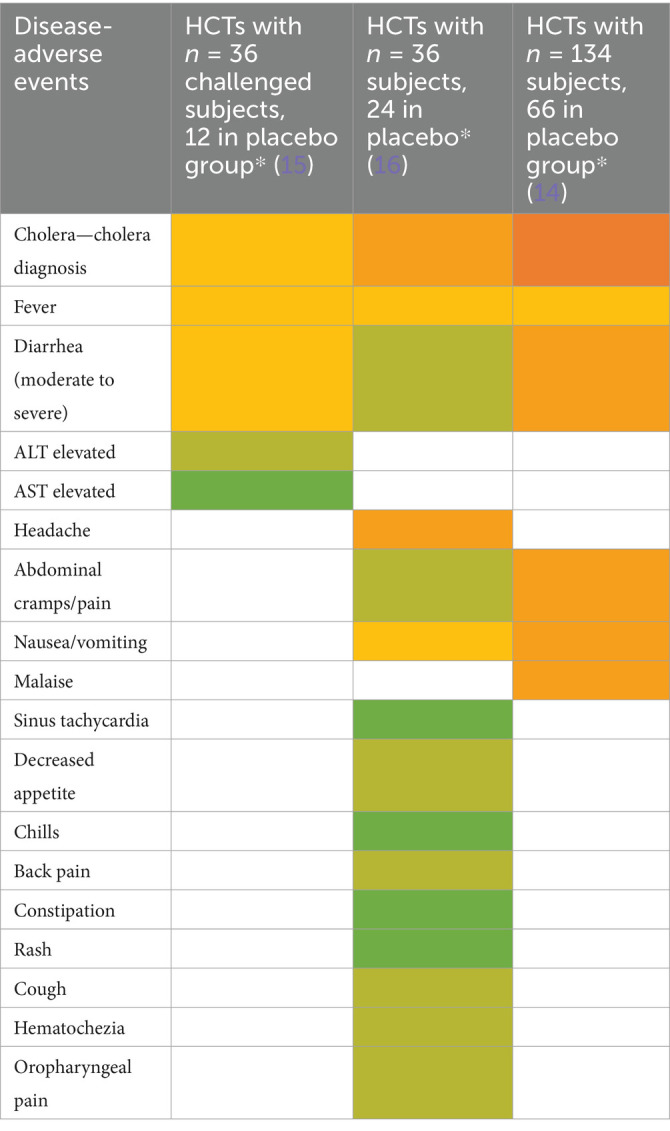
Adverse events in CHIMs for enteric infections—cholera.

**Table 3:**
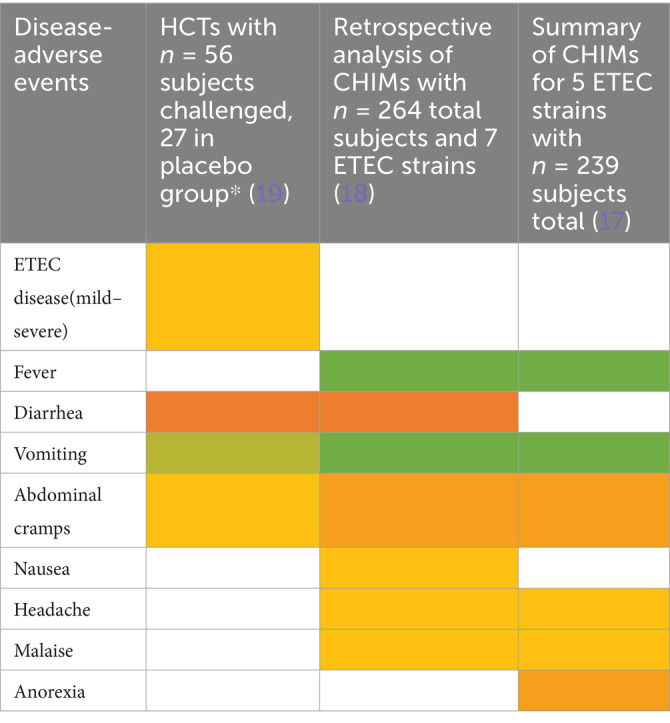
Adverse events in CHIMs for enteric infections—ETEC.

**Table 4:**
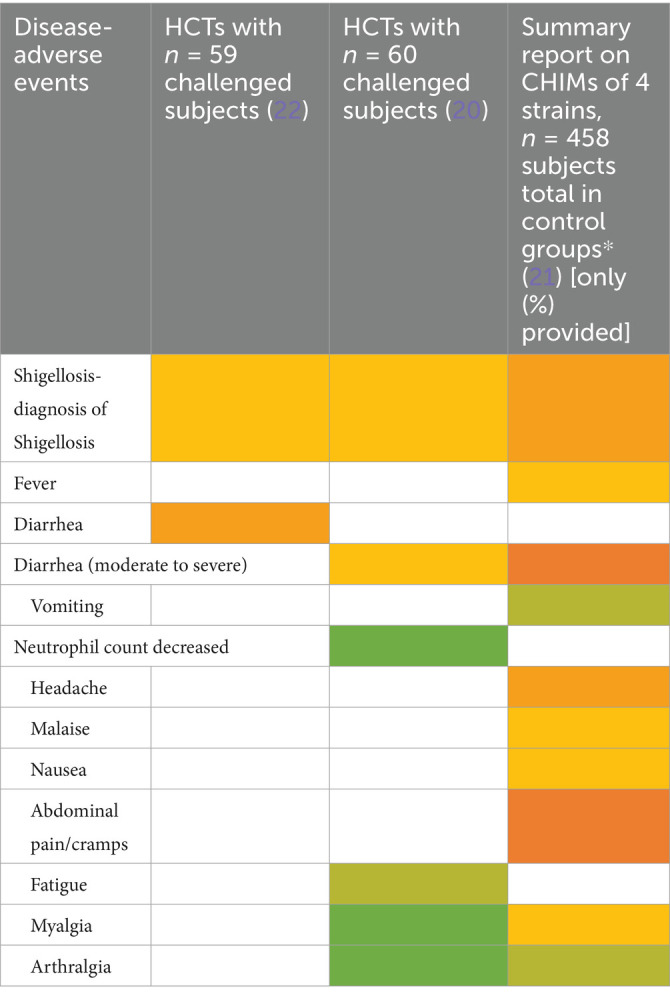
Adverse events in CHIMs for enteric infections—Shigellosis.

**Table 5:**
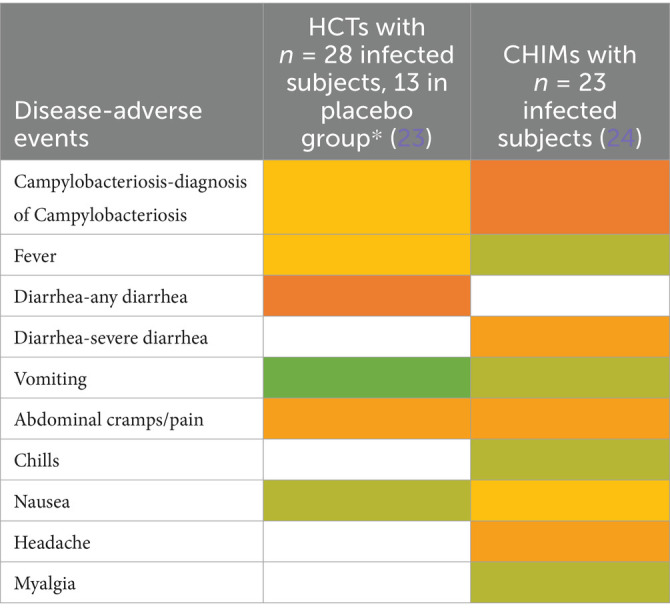
Adverse events in CHIMs for enteric infections—Campylobacteriosis.

**Table 6:**
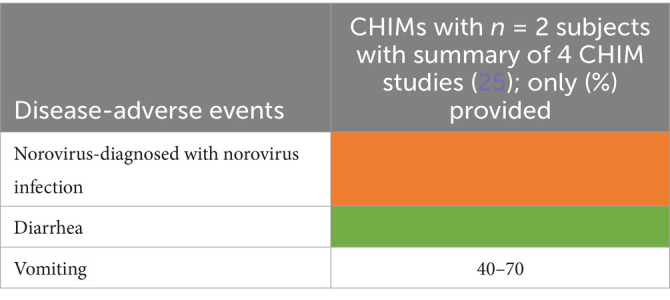
Adverse events in CHIMs for enteric infections—Norovirus.

Prediction of attack rates is essential for study design and estimation of power. However, the definition of the primary clinical endpoint varied strongly among trials. Attack rates varied, ranging from 49 to 56% in (para)typhoid CHIM, 42 to 92% for cholera, 54% in ETEC CHIM (19), 25 to 100% for shigellosis CHIM, 50 and 96% in campylobacteriosis studies, and 92% in the norovirus study. The differences in obtaining infection manifestation reflect the virulence of the challenge strain and individual predisposition, which are hard to entangle. For cholera, one study enriched for blood group O participants to assess risk and vaccine protection in the more susceptible blood group O individuals (14). Moderate-to-severe diarrhea in the unprotected control subjects was observed in 59% of the control population and in 69% of the blood group O controls. Reference is made to similar results in studies performed before the year 2000 (26–29).

The most important clinical endpoints and AEs were fever, diarrhea, and vomiting in this disease category. These three parameters were inconsistently subcategorized for severity grading. For exemplification, different fever definitions are provided in Tables 1–6 in the section on typhoid fever. Notably, fever > 40°C, which is characteristic of typhoid, was only reported in one study and one patient (13). This might be due to prophylactic medication or the choice of the challenge agent. Notably, in some studies, clinical symptoms were accompanied by laboratory abnormalities, which include imbalances and elevated liver transaminases. Further AEs such as “reduced daily activity” or “requirement for early antibiotic (or intravenous fluids)” listed in (19) are not commonly reported. However, they are indicators of the clinical burden of study participants.

### Respiratory diseases

**Figure 2:**
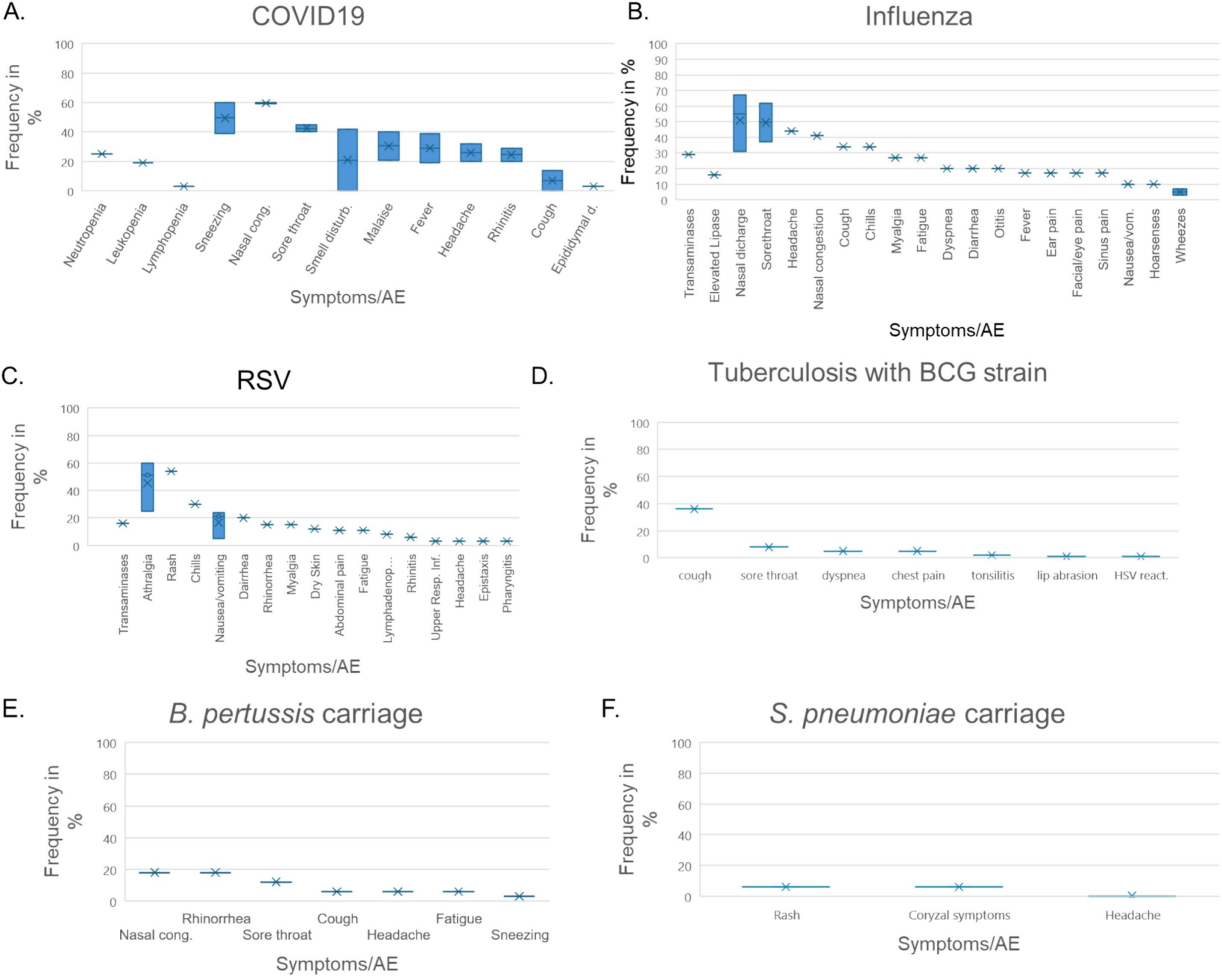
Adverse events in CHIMs and HCTs for respiratory pathogens. Reported values are given in percentages (%). A detailed list for each AEs categorized by CHIMs and references is presented in Tables 7–12. **(A–D)** Infection models for **(A)** COVID-19 (coronavirus disease of 2019); **(B)** Influenza; **(C)** RSV (respiratory syncytial virus infection); **(D)** tuberculosis with BCG strain; **(E,F)** colonization models for **(E)** *B. pertussis* carriage and **(F)** *S. pneumoniae.*

Ten studies were summarized to extract the most frequent AEs described in respiratory CHIMs. These studies include infection with viruses SARS-CoV-2 (30, 31), influenza (32, 33), RSV (34, 35), bacterial colonization studies (*Bordetella pertussis* (36), *Streptococcus pneumoniae* (37, 38)), and the *Mycobacterium bovis* BCG vaccination strain for mimicking tuberculosis (39). AEs are summarized in Tables 7–12 and are shown in Figure 2.

**Table 7:**
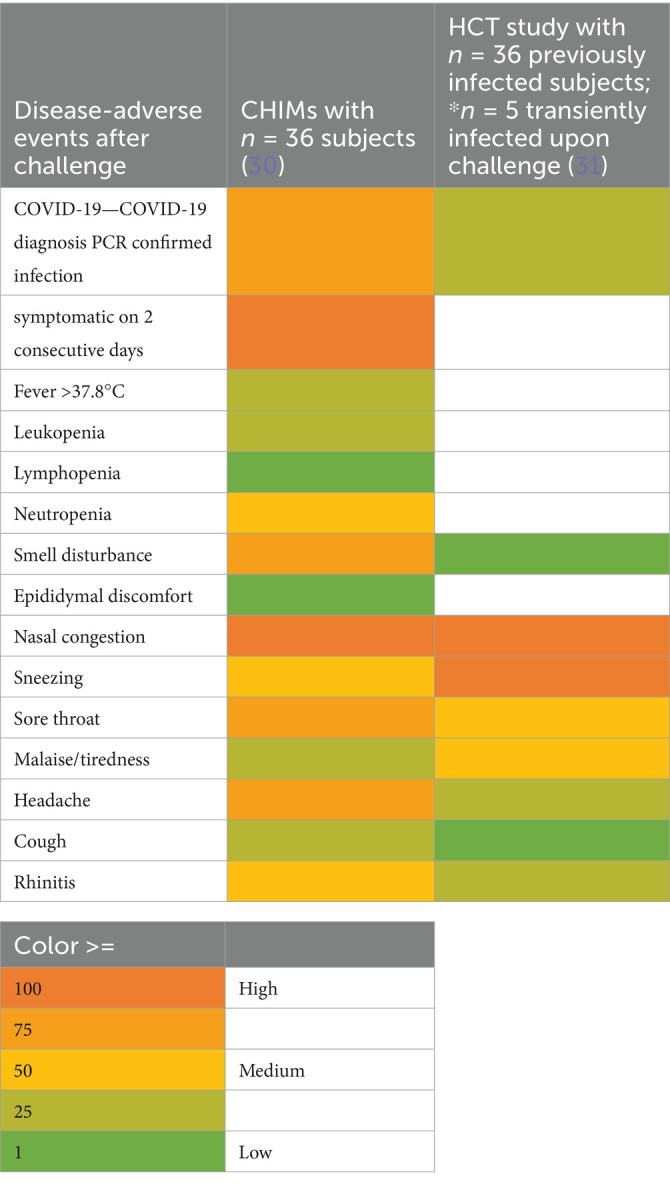
AEs documented in CHIMs and HCTs for respiratory pathogens —COVID-19.

**Table 8:**
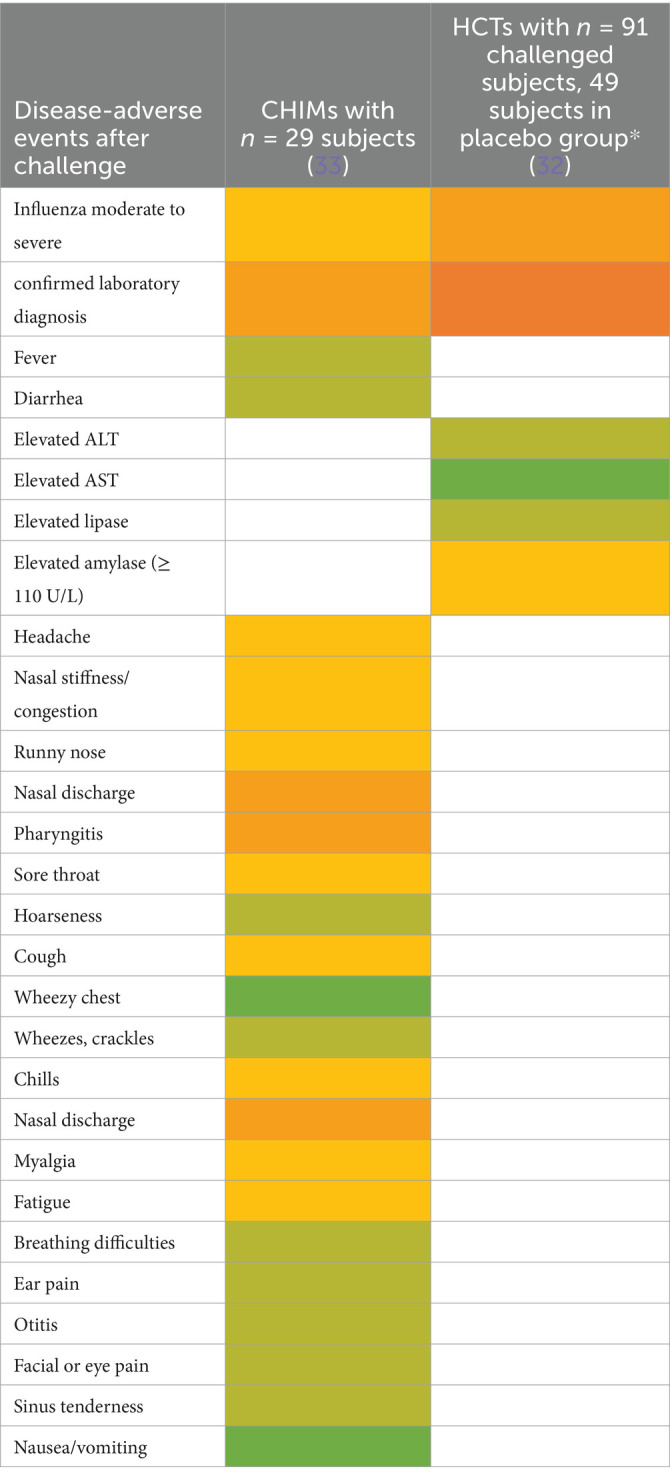
AEs documented in CHIMs and HCTs for respiratory pathogens —Influenza.

**Table 9:**
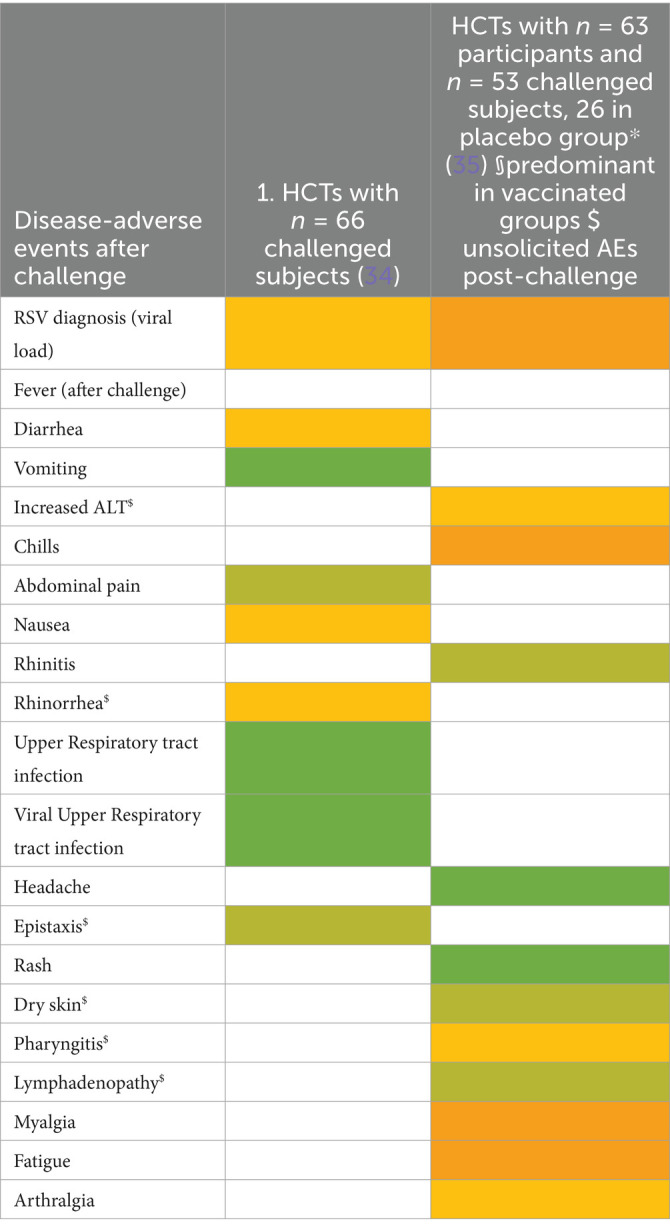
AEs documented in CHIM and HCT for respiratory pathogens—RSV.

**Table 10:**
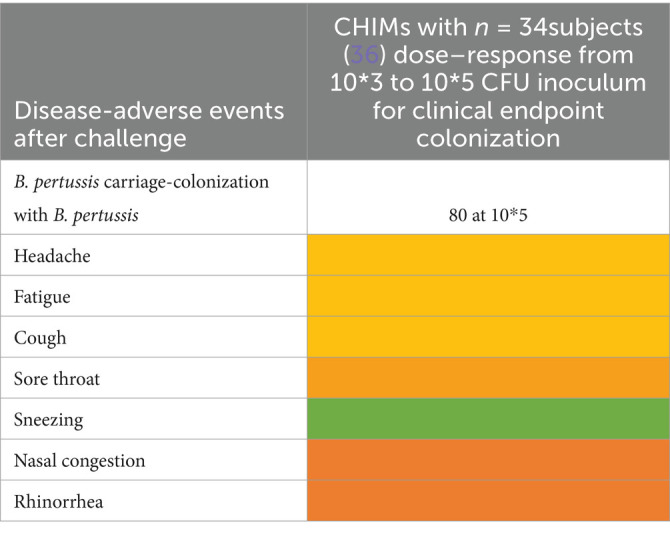
AEs documented in CHIMs and HCTs for respiratory pathogens —*B. pertussis* carriage.

**Table 11:**
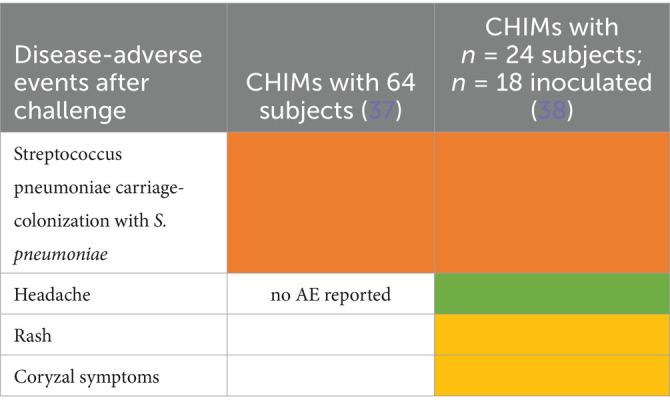
AEs documented in CHIMs and HCTs for respiratory pathogens—*Streptococcus pneumoniae* carriage.

**Table 12:**
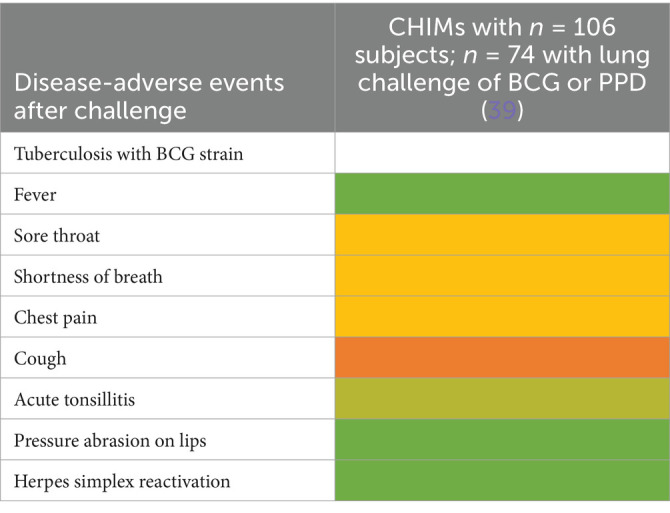
AEs documented in CHIMs and HCTs for respiratory pathogens —tuberculosis with BCG strain.

In this category, diagnosis of infection was usually defined clinically as moderate-to-severe infection and confirmed by laboratory diagnosis. The latter includes asymptomatic infections with low severity. For example, in CHIMs for influenza (32, 33), 45 or 69% of subjects were clinically diagnosed and, as expected, more (e.g., 55 and 88%, respectively) were diagnosed positive for influenza by laboratory testing. However, in CHIMs developed for COVID-19, 61% of participants were symptomatic and only 50% were positive for SARS-CoV-2 (30). In individuals with a confirmed history of infection, only 14% were transiently infected after challenge and reported symptoms, which were not specific to the challenged group (31). Notably, community-acquired SARS-CoV-2 infections were observed in 39% of volunteers (31). RSV infection was determined by viral load (53 versus 65%) (34, 35). Colonization with *B. pertussis* or *S. pneumoniae* was dependent on the inoculum size (36–38).

A detailed list of AEs observed in respiratory CHIMs can be found in Tables 7–12. Fever was detected in 19% of SARS-CoV-2-inoculated subjects and in 17% of those inoculated with influenza. Disease-typical symptoms (AEs) included smell disturbances in COVID-19-CHIM. The HCTs for RSV vaccines exemplify the difficulty of distinguishing AEs related to immunization from those induced by pathogen challenge. Despite the time interval between immunization and challenge, the data provided do not sufficiently differentiate the events, albeit a trend for more AEs in the vaccinated group is seen in (35). However, the placebo group can be used to identify challenge-related AEs. AEs in bacterial colonization studies were rare, which fits well with the absence of infection.

### Vector-borne diseases, including malaria

We next followed up 11 reports on CHIMs developed for vector-borne diseases, i.e., two on dengue fever (40–43), eight on malaria (44–51), and one study on Leishmania major (52), regarding documented AEs. In the CHIM studies for dengue, viremia was found in 85–100% (40). The most frequent AEs were rash (67–90%), headache (41–98%), and postorbital pain (35–93%), which was found only in Dengue-CHIM. Laboratory anomalies varied. For example, leucopenia reached 100% in one study and 83% in another, but was not reported in (43).

**Figure 3:**
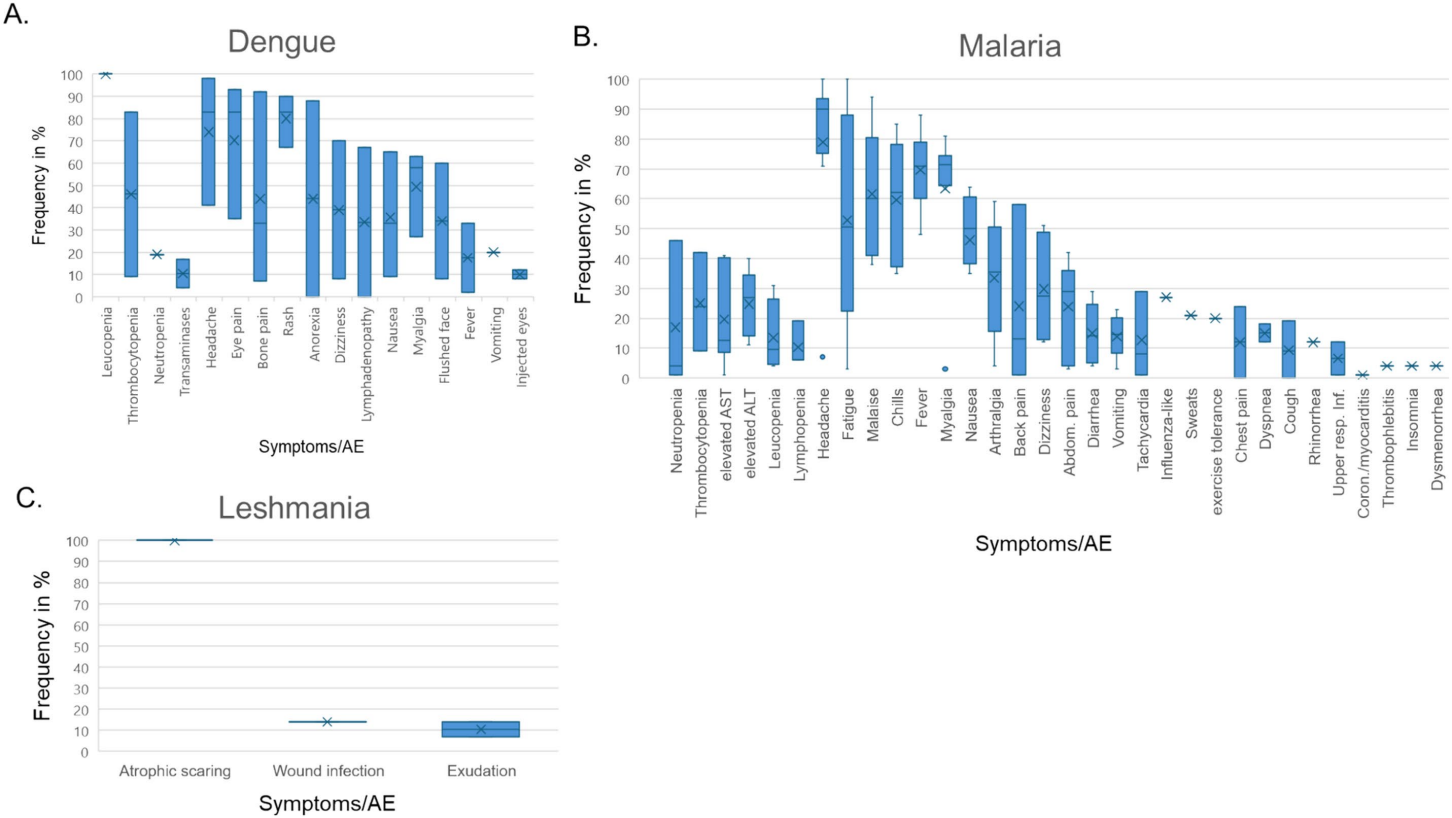
AEs in CHIMs for vector-borne diseases. The graphs summarize the reported AEs for **(A)** dengue; **(B)** malaria; **(C)** Leishmania. Frequencies are provided in percentages (%).

Clinical diagnosis of malaria and parasitemia was found in 100% with the exception of one study with 95% (49). The manifestation of malaria-typical fever in CHIM volunteers ranged from 48 to 88% (Tables 13–15). Unspecific symptoms were frequently reported but varied strongly: headache (7–100%), malaise (38–94%), fatigue (3–100%), nausea (4–64%), myalgia (3–81%), the wide range possibly reflecting differences in inoculation and the volunteer population. Among laboratory abnormalities, elevated liver transaminases were the most frequent finding, with alanine transaminase (ALT) increased in 11–40%. Recrudescence after treatment in HCTs is described in (46); a second recrudescence occurred in five subjects, but parasitemia was cleared after treatment. For more specific findings in CHIMs, see Tables 13–15 and shown in Figure 3. These include scarring and wound infections typical for the Leishmania model (52).

**Table 13:**
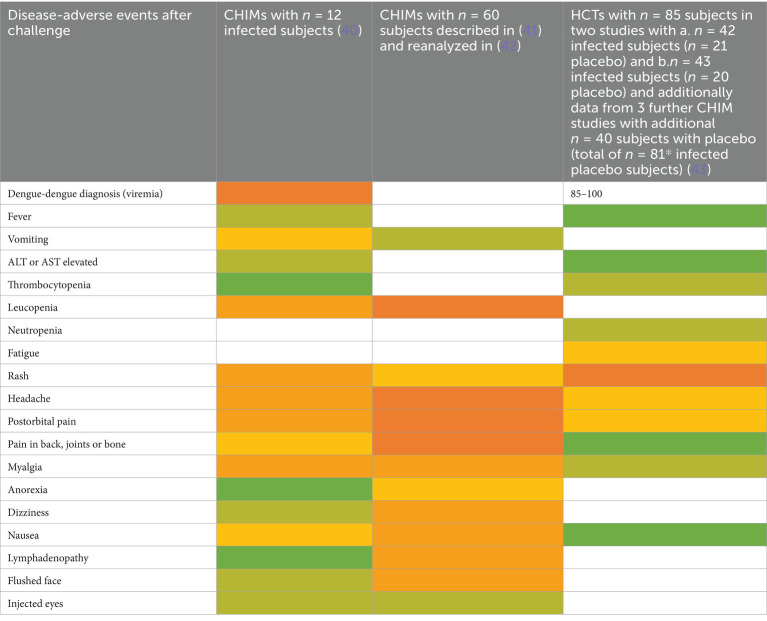
AE in CHIM for vector-borne diseases—dengue.

**Table 14:**
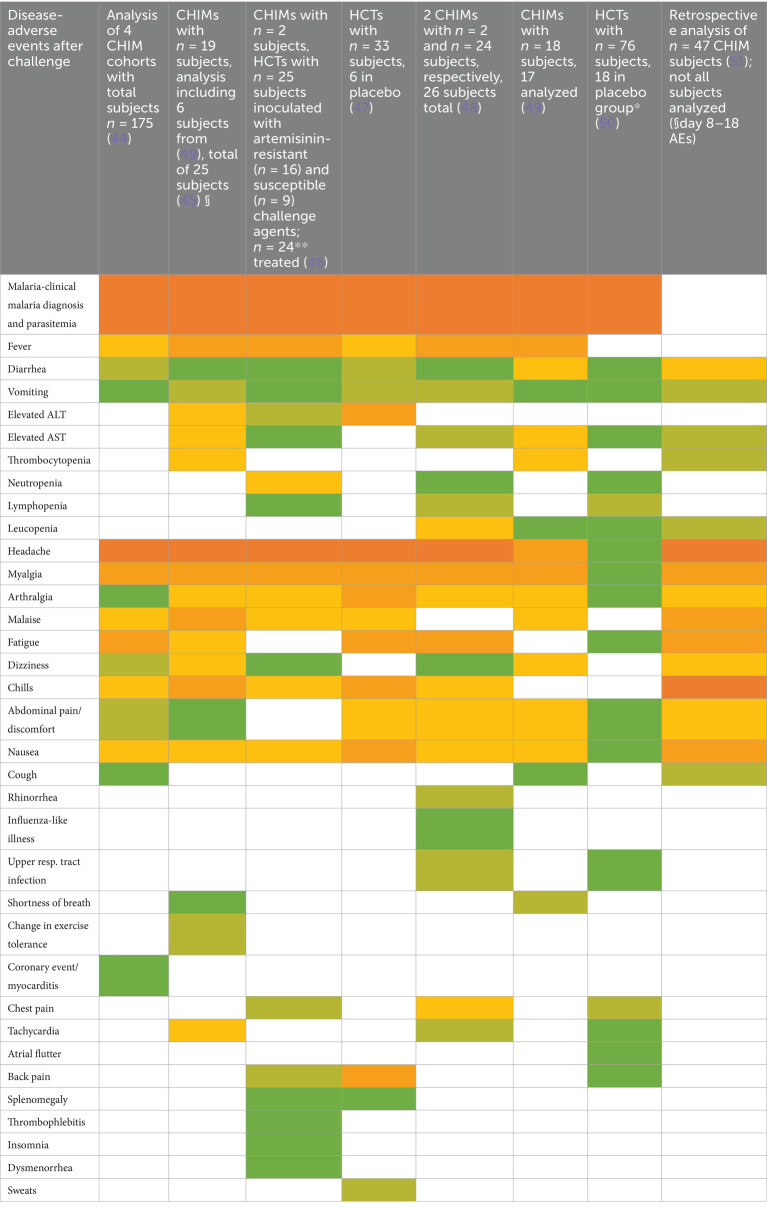
AEs in CHIMs for vector-borne diseases—malaria.

**Table 15:**
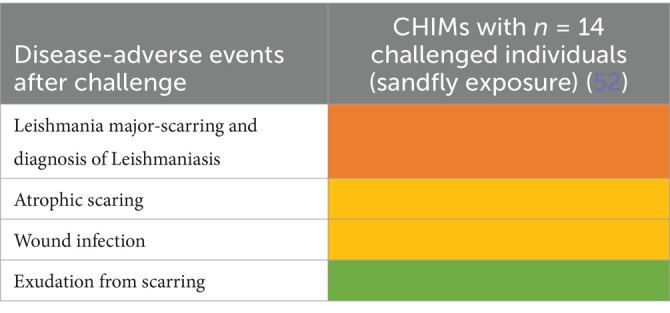
AE in CHIM for vector-borne diseases—Leishmania.

### Water- and soil-borne parasitic infections

**Figure 4:**
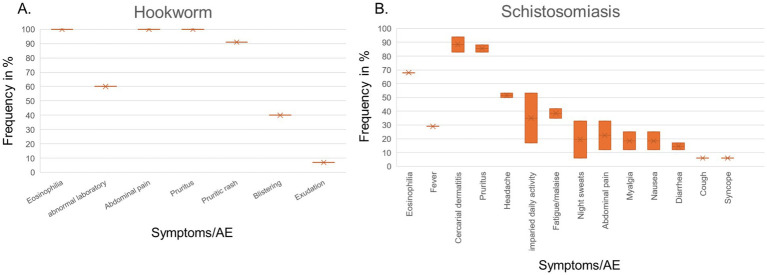
Adverse events in CHIMs of parasitosis acquired in environmental habitat. The frequencies are provided in percent (%) for hookworm **(A)** and schistosomiasis **(B)** infections. A detailed list for each AEs categorized and references is presented in Tables 16, 17.

CHIMs for two parasite infections were analyzed. For hookworm, two HCT studies were included (53, 54) that described abdominal pain as the main symptom in all subjects. Of note, blistering (6/10 (60%)) and exudation (4/10 (40%)) were observed after vaccination and are, therefore, an AE attributable to the tested vaccine, which was confirmed in 5/15 vaccinated and infected subjects (Table 16). Eosinophilia was also observed upon exposure to larvae in (55). In Schistosomiasis-CHIM (56, 57), the predominant symptoms were fever and headaches, both of which resulted in the study participants being unable to carry out their daily activities. Pruritus and cercarial dermatitis developed upon successful infection in approximately 80–94% of subjects; accompanying eosinophilia was higher in vaccinated individuals (57) (Figure 4).

**Table 16:**
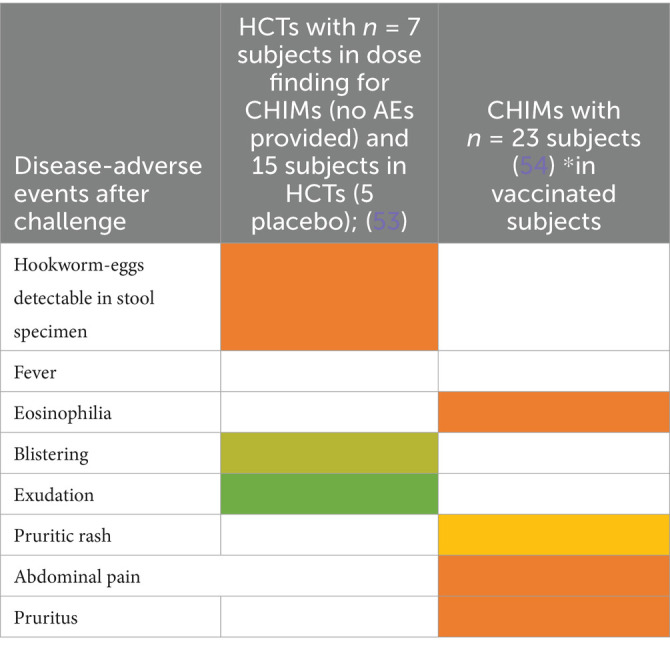
AE in parasitosis acquired in the environmental habitat—Hookworm.

**Table 17:**
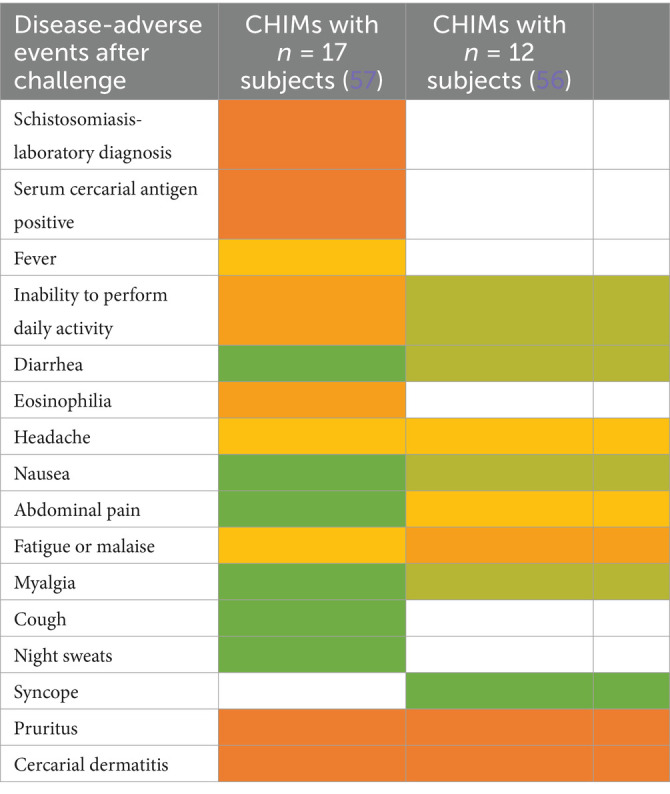
AE in parasitosis acquired in the environmental habitat—Schistosomiasis.

### Reporting of delayed adverse events

Only few studies among the screened studies reported on delayed AE CHIMs. This was due to factors such as short study duration, lack of long-term follow-up, limited sample size, and confounding factors. The reported delayed AEs have been stratified by diseases categories and are summarized presented in the Tables 18–21 below according to the diseases category and for the vaccine group, and placebo group accordingly.

**Table 18:** Delayed AEs by enteric infections.

Disease	Vaccine/placebo group	
	Delayed AEs	Outcome/Follow-up (source)
Enteric infections		
Cholera (16) ^*^	Pyelonephritis (Grade 3) ^P^*	8 Weeks after discharge (D68 after enrollment), unrelated to study treatment
ETEC (17)	Post-infectious irritable bowel syndrome^V/P*^	Workshop discussions
Shigellosis (20)	One participant ^P^, 4 SAEs: Deep vein thrombosis, pelvic venous thrombosis at D107*, hematoma at D121, and carotid artery aneurysm at D130 from challenge	All events unrelated to any study treatment and resolved
Campylobacteriosis (23)	Asymptomatic recrudescence (*n* = 2) receiving rifaximin ^V^* and (*n* = 3) placebo	D21–D56
	A second recrudescence (77 days after challenge) ^P^	ATB + Probiotic for 7 days, lost to follow-up

V, vaccine group; P, placebo group; V/P, vaccine & placebo group; D107, Day 107.

**Table 19:** Delayed AEs by respiratory pathogens.

Respiratory pathogens	Delayed AE	Outcome/Follow-up
COVID-19 (30)	At Day 180: Smell disturbance (*n* = 5) Smell impairment (*n* = 1) ^V^	6 months and after smell training advice (*n* = 6) short courses of oral and intranasal steroids (*n* = 2)
RSV (34, 35)	Mild myocarditis (*n* = 1) (↑troponin level), normal electrocardiogram (ECG) and a cardiac scan interpreted as mild myocarditis (34)^P^	no time point provided: The event resolved spontaneously
	Right ovarian cyst (*n* = 1) at 8 weeks post-challenge (35) ^V^	unrelated to study IP or challenge virus

IP, investigational product.

**Table 20:** Delayed AEs by vector-borne diseases.

Vector-borne diseases	Delayed AE	Outcome/Follow-up
Malaria (44–47, 50)	Bleeding or thrombogenic complications are not reported, but in trials where longer parasitemia is expected, platelet count monitoring should be considered (44)^V^	no time point provided: platelet count monitoring
	Thrombocytopenia, Grade 3 (*n* = 4 participants) (45)^V^	no time point provided: resolved after malaria treatment
	Ventricular extrasystoles (*n* = 1 ART-S and *n* = 1 ART-R infected participants) on D9 (46)^V^	ongoing by the EOS*
	Transient prolongation in QT interval (*n* = 4 ART-R and *n* = 1 ART-S infected participants) (46)^V^	Resolved by the EOS (pilot study, D90; comparative study, D55)
	↑Transaminases (*n* = 2) at 4 weeks PDOC * and in (*n* = 1) at 8 weeks PDOC (47)^V^	Normal transaminases upon completion of treatment
	Increase in QTcF (corrected QT interval) inconsistently (50)^V^	Up to D42 post-CHIMs, clinical relevance could not be established

EOS, end of the study; PDOC, post-day of challenge.

**Table 21:** Delayed AEs by parasitosis.

Parasitosis	Delayed AE	Outcome/Follow-up
Hookworm (53, 54)	↑ in eosinophil count occurred in all participants (among vaccine group > placebo group) (53)^V^	D161
	↑ Total IgE only in the vaccinated group (53)^V^	D1—D112
	Gastrointestinal symptoms: (*n* = 13/16), peaked at weeks 4–5, resolved at week 8 after first injection (54)^V^ (*n* = 3/16) resolved with albendazole treatment (54)^V^	Week 8 and after
Schistosomiasis (56)	Persistent infection (*n* = 4/13, all after exposure to 20 cercariae) despite multiple treatment with PZQ or artemether did not result in cure (56)^V^	At the 1-year follow-up over time 3 participants self-cured.

Ig E, immunoglobulin E; PZQ, praziquantel.

## Discussion

Development of new vaccines and anti-infectives can benefit from an established CHIM and the possibility to perform HCTs (8, 9). This became a driver for COVID-19 CHIM development in the SARS-CoV-2 pandemic (30, 31, 58, 59) and is pursued in infectious diseases where the efficacy of vaccines is difficult to assess in classical clinical trials, such as tuberculosis (39, 60). However, the relevance of data obtained in CHIMs and HCTs strongly depends on the reproducibility and the challenge agents´ mimicking of natural disease (5–7, 61), which comes at a cost for participants, which are subject to symptoms potentially interfering with daily life activities. There is currently no definition of the grading of severity of disease that is needed to provide reliable data on vaccine or drug efficacy in HCTs, and, in addition, no definition of acceptable and unacceptable risks. Thus, decision-making on the feasibility of CHIMs and HCTs is strongly dependent on a study-specific ethics approval, which is primarily based on “doing no harm,” e.g., assessing the potential safety risks for participants, thus favoring low risk and low AE profiles (2–4, 62). The inherent contradiction arising from the requirement to obtain a disease course with predictive value for natural infections remains an unresolved issue and leads to potentially inconsistent trial-specific decisions of the relevant ethics boards and regulatory bodies (2, 59, 62, 63). Moreover, most of the studies included in our analysis were conducted in upper-middle-income countries, which might have resulted in differences in reported AEs when compared to low- and middle-income countries (LMIC) where some of the infections are endemic. In addition, the higher disease burden in LMIC results in a greater need for vaccine development. Thus, there might be a requirement to conduct more CHIM studies in LMIC or countries with comparable epidemiology and socioeconomic conditions (64).

Here, we provide an overview of symptoms and AEs described in the evaluated studies pooled by disease entity, to provide a more general overview and pave the way for more general guidelines on evaluating CHIMs and HCTs. Overall, the conclusions drawn from our review indicate that symptoms and AEs correspond to those expected upon loco-typical manifestation of infection. Vector-borne and environmental uptake of parasites is also associated with typical symptoms for the pathogen and the infection route, such as scarring in Leishmaniosis, fever and chills in malaria, or eosinophilia in hookworm and schistosomiasis infections. In some CHIMs, symptoms are mitigated due to protective measures taken, such as continuous intravenous fluid and antibiotics administration in CHIMs for cholera (14–16) as well as other enteric pathogens, which serves to secure study participant safety.

Importantly, HCTs were not designed as safety studies. In addition, we cannot exclude that the occurrence of infection symptoms is masking AEs related to vaccination or a drug as long as AEs are unspecific and compatible with the infection. It is further difficult to discriminate whether ALT and AST elevations are caused by infection or treatment in malarial studies with artemisinin (45). By contrast, the blistering described in the hookworm vaccinated group in (52) is specific and noticeable. Nevertheless, the current analysis does not include sufficient data to evaluate whether AEs originating from drugs or vaccines tested in HCTs are sufficiently detected. In some cases, inadequate or incomplete data on adverse events following immunization (AEFI) can be deemed either ineligible for causality assessment or unclassifiable (65).

Notably, fever is a measurable parameter and, in many cases, reflects systemic disease manifestation as well as severity of infection. Independent of the infection, fever is the most frequently and probably most sensitive indicator described in all models. It is therefore a key parameter evaluated in all studies. When comparing malaria and dengue fever models, CHIMs for malaria report rates of nearly 48–88% while the fever rate is lower (25%) in Dengue-CHIM. Despite the low number of subjects per study, the latter most likely reflects the variability of disease manifestation in a genetically diverse population rather than the suitability of the challenge agent, and it is, of course, influenced by trial-specific criteria for medical intervention such as early-onset treatment based on positive qPCR (66).

Moreover, the manifestation of specific symptoms such as cough or hives in respiratory models is only documented in a minority of subjects. This could again be related to the reduced virulence of challenge agents and the mild course of infection in healthy volunteers. From a safety perspective, attenuated virulence of the infectious agent is advisable, but marked variation in disease manifestation can also limit the conclusions that can be drawn from the study results.

A recent report by Adams-Phipps et al. (67) performed a systematic review and meta-analysis of trial design and safety reporting in CHIMs over several decades. Despite a possible bias based on the study selection criteria in this report, our analysis confirms the observation that side effects are inadequately documented and discussed in many publications on CHIMs and HCTs. Nevertheless, Adams-Phipps et al. conclude that the overall risk profiles of HCTs and CHIMs are low. Here, we conclude that it lies in the nature of the induced infections that symptoms such as fever and diarrhea, or vomiting can impede daily activities in study subjects. The data reviewed in this study identified potentially severe AEs such as reactive arthritis in typhoid-CHIM (13), elevated AST and ALT levels in CHIMs for cholera (16), influenza (24), RSV (35), dengue (40), malaria (45, 46, 48, 50, 51), or excessive diarrhea in enteric infection models, which required medical intervention related to the infection with the challenge agent such as administration of intravenous fluids and antibiotics. In Shigella-CHIM, i.v. fluid administration was reported in 13/29 (45%) (22) and 36/60 (60%) (20), respectively, emergency room visits for hypotensive shock in 16/60 (27%) in (20), and early need for antibiotics in 18/29 (62%) in (22). Similarly, in ETEC CHIM, the authors reported requirements for i.v. fluid in 18/56 (32%) and for early antibiotics in 28/56 (50%) along with reduced daily activity in 32/56 (57%). Intravenous fluid substitution and antibiotics were also needed in 8/23 study participants (35%) in *C. jejuni*-CHIM and in 20/23 (87%), respectively (24), and administration of both fluid and antibiotics was reported in all Cholera-CHIM subjects (14–16). Notably, i.v. fluid administration was only reported in 2% (3/175) in a Malaria CHIM study (44). In view of the specific medical intervention needed, i.v. fluid and antibiotic administration is, thus, more frequent in enteric models.

These experiences further denote that the symptoms and AEs resulting from CHIMs can be medically managed and are not considered life-threatening, but can interfere with daily activities and result in significant stress. This is important because it reflects morbidity and disease burden that need to be evaluated for informed consent and ethical considerations. Notably, no deaths were reported in the evaluated studies nor mentioned by Adams-Phipps et al. (67). To improve tracking of delayed AEs in CHIM studies, extended follow-up periods, post-study surveillance studies, and real-world data integration should be considered.

Diarrhea and vomiting are characteristic of CHIMs with most enteric pathogens. Acknowledging that AEs and AE severity are disease- and in some cases pathogen-specific, recommendations for categorizing and grading AEs could alternatively be based on a syndromic approach by organ or disease type rather than with sole reference to a single challenge agent. We further observed that available guidance on severity scoring and grading was frequently adapted to serve the individual study’s purpose. For example, a retrospective reevaluation on the influence of the challenge strain on diarrheal disease severity resulted in a modified scale rather than an assessment of residual risk for volunteers (17).

Despite existing legal frameworks (such as in the EU (68, 69)) and guidance on performance of CHIMs and HCTs (70) as well as on toxicity grading and AE classification (71–73), in the current settings, comparability of data regarding the severity of AEs can therefore not be assumed and was therefore not systematically analyzed in this study. However, improved standardization of trials could provide a means to categorize AEs and define the residual risk associated with a certain type of infection. AE-informed risk–benefit assessments in CHIM design could further be considered as a basis for informed consent of subjects and support ethics committee decisions. Structured benefit/risk evaluation as provided by the European Medicines Agency (EMA) represents an important prerequisite in this research area (74).

Well-defined standards further permit the comparison of studies and thus facilitate the evaluation of a larger study population. This implies that study sites implement high standards in training and effective measures in quality management and risk mitigation strategies to secure the safety of subjects, patients, and the environment as proposed in Ref. (75). As recently proposed, specialized ethics boards and/or CHIM observers or auditors could pave the path for implementation of appropriate ethical frameworks and standards and thereby drive the development of guidance and criteria for the performance of CHIMs and HCTs (76). This comes along with the requirement to build public trust through transparent communication on potential scientific and social value and risks with the public, patients, and the medical communities (77).

## Author’s note

This report summarizes data retrieved from the publications selected for analysis. The analysis and interpretation of data reflect the conclusions drawn by the authors, but do not necessarily reflect the view of Paul-Ehrlich-Institut.
